# Peritoneal Dialysis Modality and Outcomes in the Peritoneal Dialysis Outcomes and Practice Patterns Study

**DOI:** 10.1016/j.ekir.2026.106664

**Published:** 2026-06-18

**Authors:** Thyago de Moraes, Brian Bieber, Junhui Zhao, Keith McCullough, Bruce Robinson, Ronald L. Pisoni, Tadashi Tomo, Fiona Brown, Talerngsak Kanjanabuch, Arshia Ghaffari, Simon Davies, Jeffrey Perl, Yong-Lim Kim

**Affiliations:** 1 Post-Graduate Program in Health and Biological Sciences, School of Medicine, Pontificia Universidade Catolica do Parana, Curitiba, Brazil; 2 Arbor Research Collaborative for Health, Ann Arbor, Michigan, USA; 3 Genesis Research, Inc., Hoboken, New Jersey, USA; 4 Department of Internal Medicine, University of Michigan Medical School, Ann Arbor, Michigan, USA; 5 Clinical Engineering Research Center, Faculty of Medicine, Oita University, Oita, Japan; 6 Department of Nephrology, Monash Medical Centre and Department of Medicine, Monash University, Clayton, Victoria, Australia; 7 Division of Nephrology, Department of Medicine, Faculty of Medicine, Center of Excellence in Kidney Metabolic Disorders, Chulalongkorn University, Bangkok, Thailand; 8 Department of Medicine, Keck School of Medicine, University of Southern California, Los Angeles, California, USA; 9 School of Medicine, Faculty of Medicine and Health Sciences, Keele University, Keele, UK; 10 Division of Nephrology, University of Toronto, St. Michael’s Hospital, Toronto, Ontario, Canada; 11 Department of Internal Medicine, School of Medicine, Kyungpook National University, Daegu, South Korea

**Keywords:** automated peritoneal dialysis, continuous ambulatory peritoneal dialysis, peritoneal dialysis discontinuation, peritonitis, survival

## Abstract

**Introduction:**

Automated peritoneal dialysis (APD) is the predominant peritoneal dialysis (PD) modality in many high-income countries and is increasingly adopted in low- and middle-income settings. Outcome differences between APD and continuous ambulatory PD (CAPD) remain unclear. A clearer understanding could inform clinical and policy decisions.

**Methods:**

Patients in the Peritoneal Dialysis Outcomes and Practice Patterns Study (PDOPPS; *N* = 9463 patients across 6 countries/regions) who were enrolled within 3 months of starting PD were analyzed in intent-to-treat Cox regression models examining associations between PD modality (CAPD vs. APD) and patient survival, permanent transfer to hemodialysis (HD, HDT), and time to first peritonitis, adjusting for patient and facility-level factors and stratified by country.

**Results:**

Of the patients, 87% were prescribed APD (range: 37% in South Korea to 91% in USA). APD patients were younger, had lower urine output, more often used hypertonic solutions, and less often used icodextrin. Overall, there was no evident difference between APD and CAPD in survival (adjusted hazard ratio [AHR]: 0.82, 95% confidence interval [CI]: 0.66–1.03) or HDT (AHR: 0.96, 95% CI: 0.83–1.11), though results varied by country and subgroups (APD had lower death risk in those having assisted PD). APD patients had a lower risk of peritonitis overall (AHR: 0.83, 95% CI: 0.72–0.97), and specifically of gram-positive (AHR: 0.67, 95% CI: 0.52–0.87) and culture-negative (AHR: 0.64, 95% CI: 0.44–0.95) peritonitis.

**Conclusion:**

APD and CAPD showed comparable patient and technique survival. APD’s lower peritonitis rate highlights the importance of aseptic technique; its benefits in assisted PD patients suggest that it may be suited for those requiring support.

## Graphical abstract

**Figure:**
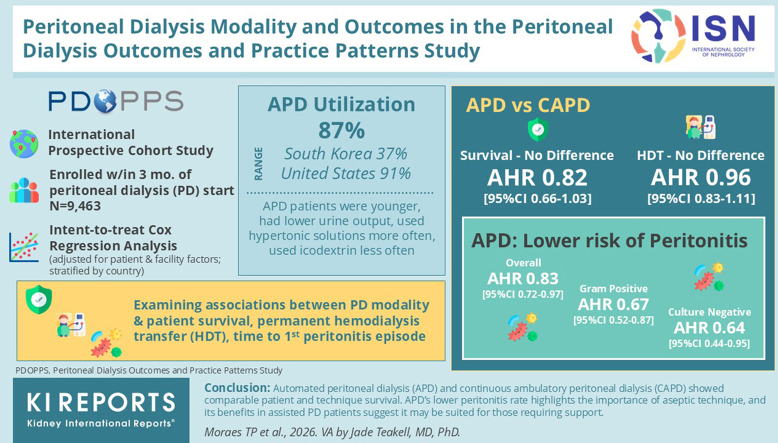

PD offers flexibility and autonomy, primarily because it can be performed at home and adapted to individual patient needs.^1^ Personalization of PD includes selecting the appropriate PD modality—APD or CAPD via shared decision making.^2^ The Kidney Disease: Improving Global Outcomes guidelines emphasize the importance of individualized PD prescriptions aimed at maintaining quality of life, preserving residual kidney function (RKF), and minimizing cardiovascular complications.^3^ Similarly, the International Society for Peritoneal Dialysis underscores that modality choice should reflect patient preferences, lifestyle, clinical circumstances, caregiver support, and local resource availability.^4^

Choosing between CAPD and APD depends on both patient preferences and clinical considerations, because the 2 modalities differ in practical aspects of treatment delivery, including timing of exchanges, treatment burden, need for equipment, caregiver involvement, and potential impact on daily activities and quality of life.^5^, ^6^, ^7^ Although APD may offer advantages for some patients, particularly with respect to daytime flexibility and alignment with work, school, caregiving responsibilities, or other activities, available evidence has not consistently demonstrated superior clinical outcomes compared with CAPD. Notably, global use of APD has increased in recent years.^8^ For example, APD prevalence among prevalent versus incident PD patients in the USA increased from 59% versus 34% to 88% versus 64% between 2002 and 2022.^9^ In Brazil, APD usage increased from 37% in 2005 to 76% in 2020, whereas Taiwan saw an increase from 11% in 2001 to 37% in 2009.^10^, ^11^, ^12^ In contrast, lower-income countries have often relied on CAPD as the primary or sole PD modality because of lower costs.^13^^,^^14^ As income levels rise, more countries may consider introducing or expanding APD to broaden patient choice and support patient-centered care goals. However, APD is associated with higher costs, and a potentially greater environmental burden and carbon footprint, underscoring the need for robust clinical evidence to inform decisions about its expanded use.^15^

Existing studies comparing PD modalities have primarily been observational and are limited in several key areas as follows: (i) they often focus solely on patient and technique survival, with fewer studies evaluating infection rates; (ii) many are small-scale, single-center, or conducted within a single country; and (iii) they frequently lack comprehensive adjustment for patient- and center-level confounders.^16^

Given these gaps, our aim was to compare HDT, overall mortality, and time to first observed peritonitis between APD and CAPD using data from PDOPPS.

## Methods

### Patients and Data Collection

PDOPPS is an international prospective cohort study of PD patients in Australia/New Zealand (A/NZ), Canada, Japan, Thailand, the UK, South Korea, and the USA conducted in collaboration with the International Society for Peritoneal Dialysis. Patients aged ≥ 18 years receiving maintenance PD were enrolled randomly from national samples of randomly selected PD facilities as described previously.^17^ Study approval was obtained from a central national institutional review board. Additional study approval and patient consent were obtained as required by national and local ethics committee regulations. Demographic, comorbidity, and treatment-related variables were collected using medical record abstraction at study enrollment. Data on clinical outcomes, including HDT, mortality, and peritonitis were collected continuously during study follow-up.

**Figure 1:**
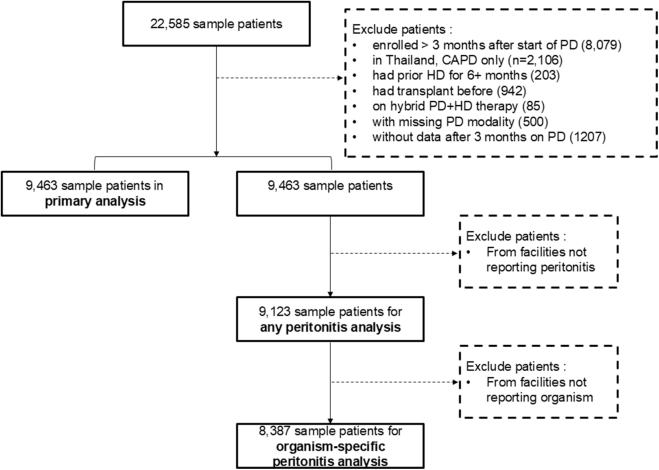
Study flow chart. CAPD, continuous ambulatory PD; HD, hemodialysis; PD, peritoneal dialysis.

The sample for this analysis included 9463 patients enrolled in PDOPPS phases 1 and 2 (2014–2021) who enrolled within 3 months of starting PD after excluding the following patients: (i) from Thailand, where APD use was minimal (5%)^18^; (ii) with >6 months of HD experience before enrolling in PDOPPS; (iii) on hybrid (HD/PD) therapy, (iv) with prior kidney transplantation; (v) missing follow-up or exposure data; or (vi) without study follow-up beyond 3 months on PD (for patients enrolled with < 3 months on PD; Figure 1).

### PD Modality Attribution

Given that in some countries, patients may initiate dialysis on one PD modality (typically CAPD), with the active intention to switch modality treatment soon after starting dialysis, we identified the intended modality for incident PD patients enrolled in PDOPPS as the modality in use at the end of 3 months after dialysis with follow-up time for outcomes starting after this 3-month time point.

### Data Analysis

The primary exposure was PD modality (APD vs. CAPD). The primary outcomes were all-cause mortality, HDT, and time to first peritonitis. Cause (organism)-specific peritonitis was explored.

We used Cox proportional hazards models to analyze the hazards of the single-event outcomes of HDT or death or first observed peritonitis. Patient time-at-risk began after modality ascertainment at 3 months on PD. Follow-up ended when reaching the particular outcome studied or at any of the following events if occurring sooner: at time of transfer out of the study facility, transplantation, death, switch to HD or to PD/HD hybrid therapy, administrative end-of-study phase, or the most recent date of data availability. However, death within 7 days of transfer to HD was counted as a death event rather than HDT. HDT was defined as a clinician-reported planned switch from PD to HD or hybrid therapy or a temporary transfer from PD to HD or hybrid therapy that did not revert within 12 weeks (84 days), with the event recorded as of initial transfer date. The primary analysis was intent-to-treat based upon the intent-to-treat modality as defined earlier. A sensitivity analysis censored patients at time of PD modality switch. Subgroup analyses were conducted by the following: (i) RKF defined as urine volume > 200 ml/d, (ii) icodextrin use, (iii) geographic region, (iv) assisted PD (caregiver involvement in exchanges), and (v) APD wet day (daytime exchange or no daytime exchanges). In addition, we performed an exploratory analysis among the small subset of individuals where peritoneal solute transfer rates (dialysate-to-plasma creatinine ratio at 4 hours) were available from a recent peritoneal equilibration test exploring outcomes by 4 peritoneal solute transfer categories (≤ 0.55, 0.56–0.64, 0.65–0.80, ≥ 0.81).

Organism-specific peritonitis was analyzed using organism categories as previously published,^19^ and without censoring when the patient experienced other organism-specific peritonitis events.

Analyses were stratified by country (the USA was further stratified by dialysis facility organization size [Large Dialysis Organization] status), adjusted for potential confounders, including patient (e.g., demographic, comorbidity, laboratory values, RKF), treatment (e.g., icodextrin, assisted PD), and clinic (e.g., facility size) characteristics. Models accounted for facility clustering using robust sandwich estimators.

For missing data, we constructed a multiple-imputation dataset with 20 replicates using IVEware.^20^ The proportion of missing data was < 10% for all imputed covariates, except for urine volume (18%). Models were estimated separately for each replicate and results were combined in standard fashion according to Rubin’s rules.^21^ Transplant waitlist, PD solution type, as well as assisted PD were not available in the data obtained from electronic health records in the US. For all analyses, we used SAS software, version 9.4 (SAS Institute, Cary, NC).

## Results

### Use of Different PD Modalities by Country

**Figure 2:**
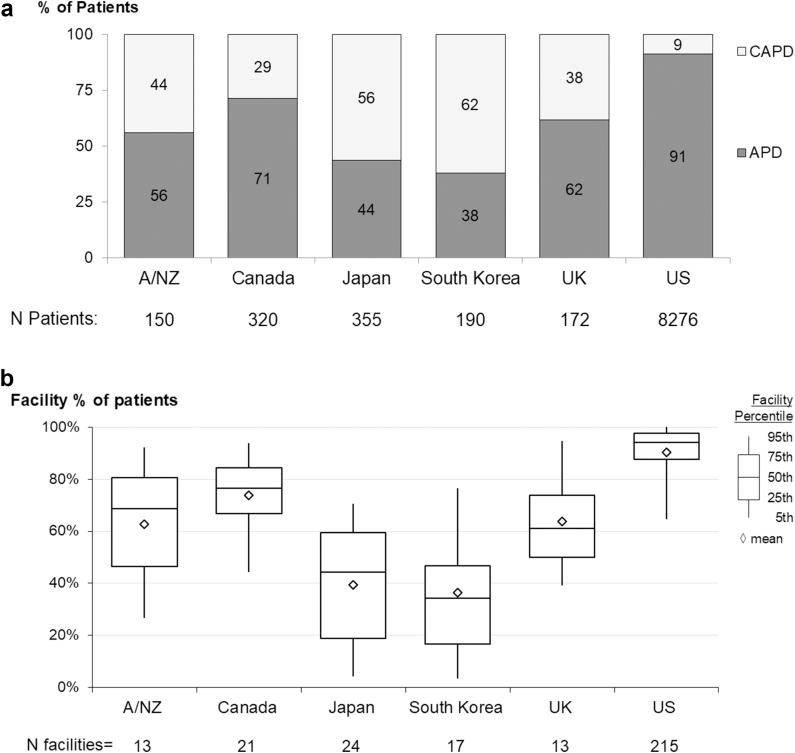
Patient-level PD modality use and facility % APD use among PD patients on PD for ≤ 3 months at study enrollment, by country. (a) Patient-level PD modality. (b) Facility percent of patients using APD at PDOPPS enrollment, restricting to facilities with ≥ 5 patients on PD ≤ 3 months at study enrollment. APD, automated peritoneal dialysis; CAPD, continuous ambulatory peritoneal dialysis.

This study included 9463 patients who enrolled in the PDOPPS within 3 months of starting PD; 1276 CAPD and 8187 APD patients, from 6 countries/regions: A/NZ (*n* = 150), Canada (*n* = 320), Japan (*n* = 355), South Korea (*n* = 190), the UK (*n* = 172) and the USA (*n* = 8276) (Figure 1). In Figure 2a, we present the distribution of PD modalities between regions, with CAPD predominant in Japan (56%) and South Korea (62%); and APD more prevalent in A/NZ (56%), Canada (71%), UK (62%), and USA (91%). In Figure 2b, we present the variation in facility percentage of patients on APD within each country. In the USA, three-quarters of the facilities had 98% of their incident PD patients on APD. APD use was less common in Japan and South Korea, where the 25th percentile of facilities had 19% or fewer patients on APD compared with the 75th percentile of facilities where ≥47% of patients were on APD.

Among the subset of incident patients enrolled in PDOPPS before 3 months on PD for whom start of follow-up was delayed until 3 months on therapy (*n* = 6025), 83% were on APD after 3 months on therapy (Supplementary Table S1). Among the patients on APD at 3 months, 21% were on CAPD initially. This pattern of initiating therapy on CAPD and then transitioning to APD within 3 months was particularly apparent in South Korea, Canada, A/NZ, and the USA. In contrast, among patients on CAPD at 3 months, a minority (6%) had started on APD.

Differences in the patient characteristics, laboratory values, and dialysis prescription between APD versus CAPD were observed (Table 1, Supplementary Table S2). Details on the connection systems are presented in Supplementary Table S2. APD patients had lower levels of RKF (based on 24-hour urine volume, particularly in the USA), had higher phosphate levels, less prescription of icodextrin. Age was similar in all countries except Japan and the UK where APD patients were younger. There were no clear differences between the PD modality groups in terms of comorbid coronary and cerebral vascular disease, heart failure, or peripheral artery disease. Hemoglobin, serum albumin, and potassium levels were also similar (Table 1).

**Table 1:** Characteristics of the clinical outcomes analysis cohorts, by PD modality among patients on PD ≤ 3 months at study enrollment

Characteristics	CAPD	APD
Number of patients	1276	8187
Demographics		
Time on PD, mos	3.5 (3.3–3.8)	3.6 (3.3–3.9)
Time with ESKD, yrs	0.35 (0.30–0.68)	0.51 (0.32–1.63)
Age, yrs	60.9 (14.8)	60.2 (15.0)
Male (%)	55%	58%
Black race (%)	11%	21%
APD wet day (%)	NA	40%
Employed^a^ (%)	33%	39%
Systolic blood pressure, mm Hg	139 (22.9)	140 (23.9)
Caregiver(s) involved in PD exchanges^a^, %	13%	17%
Comorbidities		
Coronary artery disease	16%	15%
Congestive heart failure	10%	7%
Cerebrovascular disease	5%	2%
Peripheral vascular disease	6%	3%
Other cardiovascular disease	12%	7%
Diabetes	47%	60%
Hypertension	77%	67%
Cancer (nonskin)	7%	3%
Gastrointestinal bleeding	2%	1%
Lung disease	3%	2%
Neurologic disease	2%	1%
Any psychiatric disorder	16%	20%
Recurrent cellulitis/gangrene	1%	0.70%
Transplant waitlist^a^ (%)	44%	50%
Laboratory values		
Serum phosphorus, mg/dl	5.1 (1.5)	5.6 (1.7)
Hemoglobin, g/dl	11.0 (1.5)	11.0 (1.5)
Serum albumin, g/dl	3.5 (0.5)	3.6 (0.5)
Serum potassium, mEq/l	4.3 (0.7)	4.3 (0.6)
Serum creatinine mg/dl	7.2 (3.2)	8.1 (3.9)
24-hour urine volume, l	1.1 (0.6–1.6)	0.8 (0.4–1.4)
4-hour Dialysate-to-Plasma creatinine ratio^b^	0.70 (0.60–0.79)	0.64 (0.55–0.73)
≤ 0.55	15%	27%
0.56–0.64	19%	26%
0.65–0.80	46%	36%
≥ 0.81	20%	12%
Dialysis prescription details		
Icodextrin use^c^ (%)	38%	7%
Icodextrin and high glucose solution^a^^,^^d^ (%)	12%	11%
High calcium^a^ (%)	49%	9%
Neutral pH low GDP (%)	49%	6%
Use of hypertonic glucose^a^^,^^d^		
Without any 2.27% or 3.86% use	44%	42%
Use of 2.27% but not 3.86%	45%	47%
Use of any 3.86%	11%	10%

APD, automated PD; CAPD, continuous ambulatory PD; ESKD, end-stage kidney disease; IQR, interquartile range; LDO, Large Dialysis Organization; PD, peritoneal dialysis.

Results shown as mean (SD), median [IQR] and proportion.

a Data are not available for US LDO data sources.

b Data only available for 21% of patients.

c Data only available for one of US LDO data sources.

d High-glucose solution is defined as > 50% of volume using 2.27% solution, or any use of 3.86% solution. Only available for 14% of patients.

### Patient Mortality

**Figure 3:**
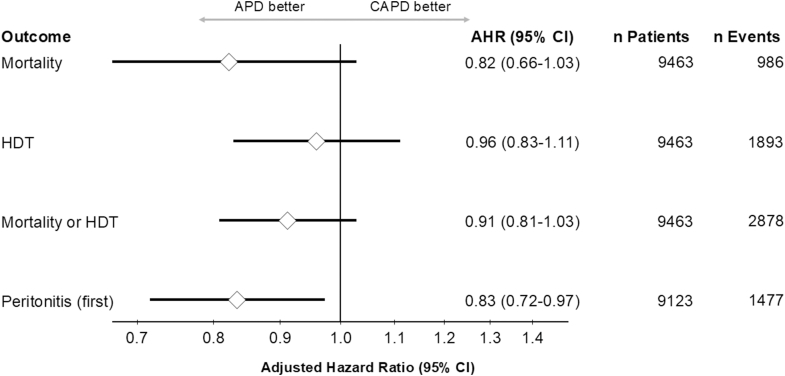
Adjusted hazard ratios of APD versus CAPD for clinical outcomes. Separate models were conducted for each outcome. Models were adjusted for time on dialysis, age, sex, Black race, 13 comorbidities (coronary artery disease, cancer [nonskin], other cardiovascular disease, cerebrovascular disease, congestive heart failure, diabetes, gastrointestinal bleeding, hypertension, lung disease, neurologic disease, psychiatric disorder, peripheral vascular disease, and recurrent cellulitis/gangrene), albumin, creatinine, 24-hour urine volume, potassium, icodextrin, transplant waitlist status, assisted PD, facility size; stratified by country; models accounted for facility clustering using a robust sandwich estimator. AHR, adjusted hazard ratio; APD, automated peritoneal dialysis; CAPD, continuous ambulatory peritoneal dialysis; CI, confidence interval; HDT, permanent transfer to hemodialysis.

**Figure 4:**
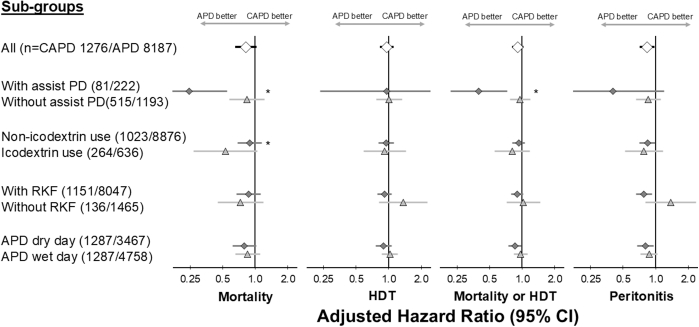
Adjusted hazard ratios of APD versus CAPD for clinical outcomes, by subgroups. Models were adjusted for time on dialysis, age, sex, Black race, 13 comorbidities (coronary artery disease, cancer [non-skin], other cardiovascular disease, cerebrovascular disease, congestive heart failure, diabetes, gastrointestinal bleeding, hypertension, lung disease, neurologic disease, psychiatric disorder, peripheral vascular disease, and recurrent cellulitis/gangrene), albumin, creatinine, 24-hour urine volume, potassium, icodextrin, transplant waitlist status, assisted PD, facility size; stratified by country; models accounted for facility clustering using a robust sandwich estimator. RKF is residual kidney function defined as urine volume > 200 ml/dl. One of the US LDOs was excluded in the subgroup analysis by icodextrin because of lack of data. US LDOs were excluded from assisted PD analysis because of lack of data. ∗ Indicates interaction *P*-value < 0.05. Underlying data available in supplementary materials. AHR, adjusted hazard ratio; APD, automated PD; CAPD, continuous ambulatory PD; CI, confidence interval; HDT, permanent transfer to hemodialysis; LDO, Large Dialysis Organization; PD, peritoneal dialysis.

During the study 986 patients died, including 839 APD patients and 147 CAPD patients. Overall APD (vs. CAPD) had no evident difference in mortality (AHR: 0.82, 95% CI: 0.66–1.03; Figure 3). When stratified by country, the estimates of AHR of mortality for APD versus CAPD ranged from 0.25 (95% CI: 0.09–0.65) in Japan to 0.96 (0.72–1.29) in the USA (Supplementary Figure S1, *P* for interaction = 0.01). AHR of mortality for APD versus CAPD use was 0.53, (95% CI: 0.27–1.05) among icodextrin users, and 0.89 (95% CI: 0.69–1.15) among non-icodextrin users (*P* for interaction = 0.04, Figure 4, Supplementary Table S3). The AHR of mortality for APD versus CAPD use was 0.24 (95% CI: 0.11–0.55) among patients having assisted PD, versus 0.84 (0.58–1.23) among patients without assisted PD (*P* for interaction = 0.004). Substantial mortality differences were not observed for APD versus CAPD users among patients with versus without RKF (*P* for interaction = 0.43), or APD wet day versus dry day (*P* for interaction = 0.56). Mortality risks were lower for APD than CAPD patients among those with dialysate-to-plasma creatinine ratio values of 0.65 to 0.80 (AHR: 0.68 [95% CI: 0.35–1.32]) and dialysate-to-plasma creatinine ratio values ≥ 0.81 (AHR: 0.40 (95% CI: 0.16–1.04); however, the interaction *P* values across solute transfer categories were not significant (*P* for interaction = 0.4; Supplementary Table S4).

### HDT

HDT during the study occurred in 1893 patients, of which 253 were CAPD patients and 1640 were APD patients. Overall, AHR for HDT transfer for APD versus CAPD users was 0.96 (95% CI: 0.83–1.11; Figure 3). Similar results were obtained when censoring patients at time of PD modality switch (Supplementary Table S5). The main causes of HDT were similar between APD and CAPD patients (*P* = 0.6; Supplementary Figure S2) with infection being the most common reason (35%). Other common (> 10% overall) causes were solute clearance, water removal problems, and psychosocial/medical causes.

### Patient Mortality or HDT

There was no association of PD modality with the composite outcome of either having permanent HDT or death (AHR: 0.91, 95% CI: 0.81–1.03; Figure 3). Our findings were consistent across patient subgroups (Figure 4).

### Peritonitis

At least 1 peritonitis occurred during the study in 1477 patients, of which 238 were CAPD patients and 1239 were APD patients. Overall, peritonitis rates were lower in APD than in CAPD users (AHR of first peritonitis: 0.83, 95% CI: 0.72–0.97; Figure 3). In organism-specific analyses, the risks were particularly lower among APD than in CAPD users for gram-positive (AHR: 0.67, 95% CI: 0.52–0.87) and culture-negative episodes (AHR: 0.64, 95% CI: 0.44–0.95; Table 2).

**Table 2:** Adjusted hazard ratio (95% CI) of APD versus CAPD for organism-specific peritonitis outcomes

Organism type	Number of events	APD vs. CAPD (AHR, 95% CI)
Gram-positive	379	0.67 (0.52–0.87)
Gram-negative	130	1.49 (0.92–2.41)
Culture negative	252	0.64 (0.44–0.95)
Fungal	9	0.43 (0.06–2.85)
Enteric	161	1.05 (0.74–1.49)

AHR, adjusted hazard ratio; APD, automated peritoneal dialysis; CAPD, continuous ambulatory peritoneal dialysis; CI, confidence interval.

*N* = 8387 patients in organism specific analysis.

Separate models were conducted for each type of organism-specific peritonitis; Models were adjusted for time on dialysis, age, sex, Black race, 13 comorbidities (coronary artery disease, cancer [non-skin], other cardiovascular disease, cerebrovascular disease, congestive heart failure, diabetes, gastrointestinal bleeding, hypertension, lung disease, neurologic disease, psychiatric disorder, peripheral vascular disease, and recurrent cellulitis/gangrene), albumin, creatinine, 24-hour urine volume, potassium, icodextrin, transplant waitlist status, assisted PD, facility size; stratified by country; models accounted for facility clustering using a robust sandwich estimator.

Overall, results were similar for all outcomes when censoring patients at time of modality switch (Supplementary Table S5, 3% of APD and 28% of CAPD patients at baseline switched dialysis modality during study follow-up).

## Discussion

In this multinational prospective cohort study that explored the association between PD modalities and clinical outcomes adjusted for numerous confounders, we found no significant differences in patient survival or HDT between patients on APD and CAPD; however, peritonitis risks were higher with CAPD overall.

Our findings are supported by previous studies that have explored the impact of PD modality on patient and technique survival. Across 3 randomized controlled trials summarized in a Cochrane review,^22^ across 139 patients, no significant differences in patient and technique survival were noted; however, these studies remained significantly underpowered and limited by relatively short follow-up.

Looking to larger observational cohort studies, in the US Renal Data System, Mehrotra et al.^1^ explored PD modality and outcomes across over 60000 incident US patients and saw no difference in adjusted mortality risk by dialysis modality but unadjusted analyses favored a lower proportion of HDT in APD patients noting its preferential use in younger healthier patients. In the Dutch NECOSAD study, after adjustment, no differences in survival were observed by dialysis modality.^23^ In the Australia and New Zealand Dialysis and Transplant registry, patient survival was examined by dialysis modality stratified by peritoneal membrane solute transfer status, finding that among slow solute transfer rates, superior survival was observed among CAPD compared with APD; however, similar to our findings, individuals with rapid solute transfer had a survival advantage in APD postulated to be because of better volume management with APD among these patients.^24^

Among patients receiving assisted PD, we found that patients treated with APD had a lower mortality risk, with no significant increase in the risk of HDT over the study period. Models of assisted PD delivery vary widely, in terms of scope of PD modality provided^25^; and in many cases, the assistants in our cohort were primarily family members or care partners. The lower mortality risk we observed may have been mediated, at least in part, by the lower risk of infection associated with APD in our study. However, given the small sample size in this subgroup, these findings should be interpreted with caution. Frailty and comorbid illness often track with the need for assisted PD. As a result, it may be more relevant to examine patient and care partner experience measures comparing assisted APD with CAPD, particularly in domains related to treatment burden and quality of life of both patient and care partner, which were not explored in the present study.

One of the novel aspects of our study has been to explore the impact in the subgroup of patients treated with icodextrin. In doing so, we observed that icodextrin users on APD had a trend toward improved patient survival compared to icodextrin users on CAPD. Previous PDOPPS data show that icodextrin use is more common among patients with less RKF, faster peritoneal solute transfer rates. and lower ultrafiltration capacity.^26^ These findings suggest that in a subset of patients who may be struggling with volume management and were prescribed icodextrin, there was an additive and potentially synergistic benefit of providing APD. This finding will need to be confirmed in prospective interventional studies.

In PDOPPS, PD modality use varied significantly across and within countries. These practice patterns are likely driven by clinical factors; socioeconomic differences across countries, culture, and/or level; and/or patient preference. In our cohort, PD modality switches during the first 3 months of therapy were largely CAPD to APD with only 6% of patients switching from APD to CAPD (Supplementary Table S1).

We found that the subgroup of patients from Japan and South Korea, in contrast to A/NZ, Canada, UK, and USA, had better patient survival on APD. This difference may relate to patient-level characteristics, including the clinical indication for APD in Japan. Compared with Japanese CAPD patients, APD patients were twice as likely to be on the transplant waiting list in Japan (28% vs. 14%) and in South Korea (78% vs. 72%) which is usually an indication of better health. This suggests differences in indication for APD perhaps dictated by a more robust and healthier group of patients who choose APD because of the ability to better maintain employment and vocational abilities.

The distributions of the main causes of HDT were similar between APD and CAPD patients but dominated by infection as the main cause in both groups, suggesting that the similar risk of HDT between APD and CAPD was not specific to any 1 cause.

We found a lower risk of peritonitis in APD than in CAPD. Our study is unique in that we had further details on the microbiology of peritonitis events and found that the lower risk in APD versus CAPD was particularly driven by lower rates of gram-positive and culture negative peritonitis episodes. Our findings were consistent across countries. Taken together, mechanistically, and consistent with other studies, this would support the hypothesis that APD may offer advantages that reduce infection risks, including fewer daily connection events and less touch contamination opportunities,^2^ which would preferentially reduce predominantly gram-positive episodes.^27^ However, systems involve more tubing, which increases surface area for potential contamination. The patient remains connected to the cycler overnight, which increases the duration of vulnerability especially in households with pets. Night-time disconnections and disconnection procedures, particularly when patients are sleepy or urgently need to disconnect, may lead to lapses in aseptic technique.

Some multicenter studies such as the Australia and New Zealand Dialysis and Transplant registry^28^ have suggested no significant difference in peritonitis rates between APD and CAPD. However, in a recent study by Cheng *et al.*^29^ using electronic medical records from the Hong Kong Hospital Authority (2007–2019) and applying inverse probability of treatment weighting in Cox proportional hazards models, the authors showed that CAPD was associated with a higher peritonitis risk than was APD. This large population-based study supports the hypothesis that APD may reduce peritonitis risk, though results are likely influenced by confounding and may not be generalizable to other regions where practice differences vary and often have less stringent and restrictive criteria for APD use than in Hong Kong. We were somewhat surprised to observe that the lower peritonitis risk in APD was not magnified in our sensitivity analysis that censored patients at a switch in PD modality. However, reducing the number of peritonitis events in this analysis may have limited our statistical power to observe a difference in this sensitivity analysis.

The strengths of the current findings include the use of a large, multinational population in which the impact of PD modality could be assessed with adjustments for a robust and comprehensive set of patient comorbidities, laboratory parameters, and demographic variables, including the exploration of subgroup analyses. However, there are some notable limitations. It is possible that decisions surrounding PD modality selection may vary according to regional policies and be influenced by differences in patient characteristics leading to potential use of APD as a rescue therapy. Therefore, residual confounding based on unmeasured factors may have affected our findings. In addition, we started follow-up for clinical outcomes after 3 months on PD to allow stabilization of PD modality assignment. Therefore, our findings are only relevant to patients remaining on PD for 3 months. However, among patients without follow-up after 3 months, only 6% died or transferred to HD. The remaining patients were administratively censored before reaching 3 months on PD under study follow-up. As such, the impact on our outcome model estimates should be minimal. Although we conducted subgroup analyses based on peritoneal solute transfer rates, this was among a very limited number of individuals among whom these were available.

Notwithstanding these limitations, in this large multinational prospective cohort study, we found no evident differences in overall patient or technique survival between CAPD and APD users, reinforcing previous evidence that these modalities provide comparable long-term outcomes. However, APD was associated with a lower risk of peritonitis—particularly for gram-positive and culture-negative episodes—suggesting a potential advantage in infection prevention. In addition, our findings highlight the nuanced role of APD in certain subgroups, such as in icodextrin users who may have greater membrane dysfunction and where APD may offer improved volume management and in those receiving assisted PD. Although regional variations and unmeasured confounders may influence modality selection and outcomes, these results support a personalized approach to PD modality choice, with APD offering distinct benefits in selected patient populations. Future prospective studies are warranted to further explore these associations, particularly in resource-limited settings and particularly with regards to peritonitis risks.

## Disclosure

AG reports being a speaker with Vantive, a speaker and consultant with DaVita, and a speaker with Akebia. BB, KM, and RLP are employees of Arbor Research Collaborative for Health**,** which receives funding for the global PDOPPS study. BR reports consultancy with Novo Nordisk; research funding from AstraZeneca; and advisory or leadership roles, including Editorial Board, American Journal of Kidney Diseases; Board of Directors, Kidney Health Initiative. JP reports ownership interest in I-Ren; consultancy with Baxter (Vantive) Health Care Canada, Bayer, Otsuka, Fresenius Medical Care, Davita Healthcare Partners, I-Ren, AstraZeneca, Outset Medical, and US Renal Care; research funding from Arbor Research Collaborative for Health and AHRQ; honoraria: Baxter Healthcare USA/Canada, Davita Healthcare Partners, Fresenius Medical Care, DCI, AstraZeneca, US Renal Care, Bayer Canada, Otsuka, and Innovative Renal Care; Speakers Bureau with Baxter Healthcare and Fresenius Medical Care; salary support from Arbor Research Collaborative for Health and AHRQ; and stipend from ISPD and ASN. TK has received consultancy fees from VISTERRA and AstraZeneca as country investigators and is a recipient of funding from the National Research Council of Thailand; and has received speaking honoraria from AstraZeneca, Baxter Healthcare, and Fresenius Medical Care. SD is on the steering committee of the CSL300_2301 POSIBIL6 trial and the Advisory Board for Byonycs. TM has received consultancy fees from AstraZeneca, Bayer, Baxter/Vantive, Boehringer, CSL Vifor, Lilly, Novo Nordisk, and Pfizer; and has received speaking honoraria from AstraZeneca, Bayer, Baxter/Vantive, Boehringer, CSL Vifor, Lilly, Libbs, Merck, Novo Nordisk, Servier, and Takeda. YK has received research funding and speaking honoraria from Baxter Healthcare. All the other authors declared no competing interests.

## Supplementary Material

Supplementary File (PDF) Figure S1. Adjusted hazard ratios of APD versus CAPD for clinical outcomes, by country/region among patients on PD ≤ 3 months at PDOPPS enrollment. Figure S2. Reason for permanent transfer to HD, by PD modality. Table S1. Modality changes before 3 months on PD among patients where follow-up delayed until 3 months on PD, by modality at 3 months and country. Table S2. Characteristics of the clinical outcomes analysis cohorts, by PD modality and country among patients on PD ≤ 3 months at study enrollment. Table S3. Adjusted hazard ratios of APD versus CAPD for clinical outcomes, by subgroups; underlying data for Figure 4. Table S4. Adjusted hazard ratios of APD versus CAPD for clinical outcomes, by 4-hour dialysate-to-plasma creatinine ratio. Table S5. Adjusted hazard ratios of APD versus CAPD for clinical outcomes censoring follow-up at PD modality switch.
