# Supplementary material for: Peritoneal Dialysis Modality and Outcomes in the Peritoneal Dialysis Outcomes and Practice Patterns Study
Source: Kidney Int Rep. 2026 Jun 18;11(9):106664. doi: 10.1016/j.ekir.2026.106664 (PMC13425579; doi:10.1016/j.ekir.2026.106664)
Supplement: Supplementary File (PDF) — Figure S1. Adjusted hazard ratios of APD versus CAPD for clinical outcomes, by country/region among patients on PD ≤ 3 months at PDOPPS enrollment. Figure S2. Reason for permanent transfer to HD, by PD modality. Table S1. Modality changes before 3 months on PD among patients where follow-up delayed until 3 months on PD, by modality at 3 months and country. Table S2. Characteristics of the clinical outcomes analysis cohorts, by PD modality and country among patients on PD ≤ 3 months at study enrollment. Table S3. Adjusted hazard ratios of APD versus CAPD for clinical outcomes, by subgroups; underlying data for Figure 4. Table S4. Adjusted hazard ratios of APD versus CAPD for clinical outcomes, by 4-hour dialysate-to-plasma creatinine ratio. Table S5. Adjusted hazard ratios of APD versus CAPD for clinical outcomes censoring follow-up at PD modality switch. [file mmc1.pdf]

Figure S1. Adjusted hazard ratios of APD vs. CAPD for clinical outcomes, by country/region

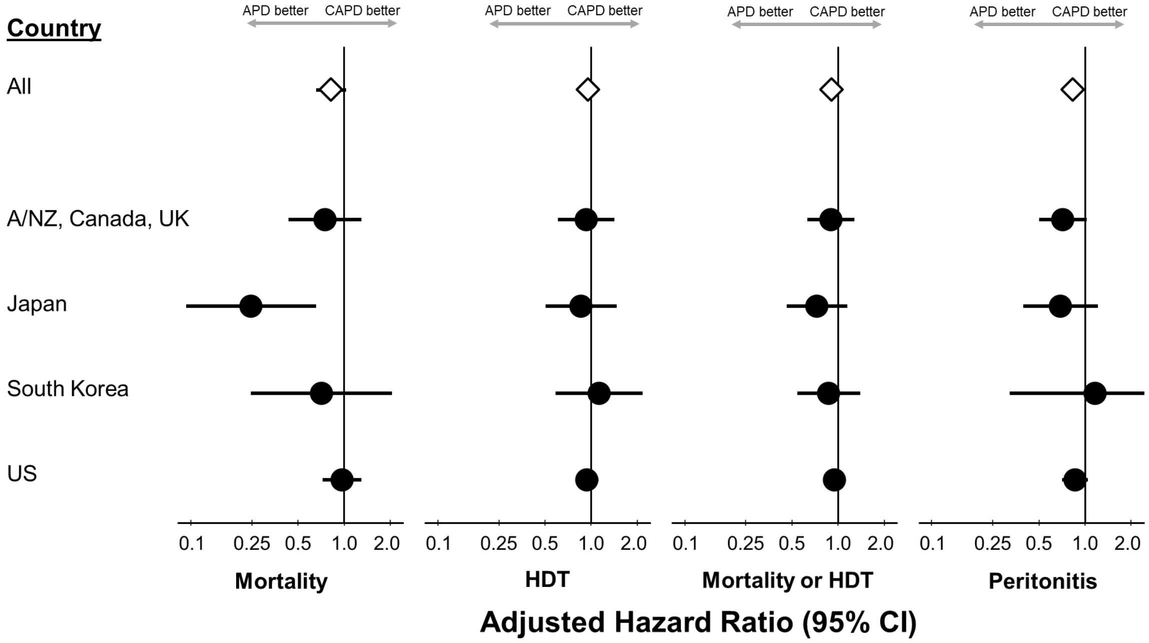

Separate models were conducted for each country and outcome. Models were adjusted for time on dialysis, age, sex, Black race, 13 comorbidities (coronary artery disease, cancer [non-skin], other cardiovascular disease, cerebrovascular disease, congestive heart failure diabetes, gastrointestinal bleeding, hypertension, lung disease, neurologic disease, psychiatric disorder, peripheral vascular disease, and recurrent cellulitis/gangrene), albumin, creatinine, 24 hour urine volume, potassium, icodextrin, transplant waitlist status, assisted PD, facility size; stratified by country; models accounted for facility clustering using a robust sandwich estimator.

Figure S2. Reason for permanent transfer to HD, by PD modality

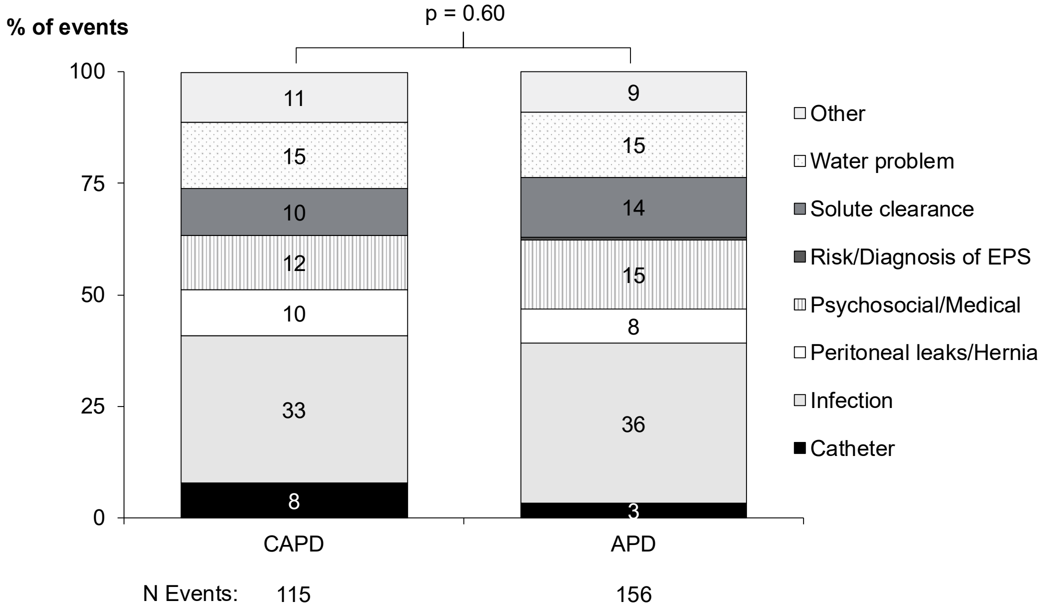

EPS=Encapsulating Peritoneal Sclerosis; Data not available for US LDO patients (n=2361 with transfer to HD). Among the remaining n=271 patients with a transfer to HD, the reason for transfer was available for 91% of cases.

Supplemental materials

Table S1. Modality changes before 3 months on PD among patients where follow-up delayed until 3 months on PD, by modality at 3 months and country

	APD at 3 months (n=4987)			CAPD at 3 months (n=1038)		
**Country**	**APD throughout**	**CAPD initially**	**n patients**	**CAPD throughout**	**APD initially**	**n patients**
**A/NZ**	62%	38%	79	95%	5%	64
**Canada**	55%	45%	241	99%	1%	93
**Japan**	93%	7%	163	96%	4%	198
**South Korea**	54%	46%	87	97%	3%	150
**UK**	96%	4%	107	95%	5%	75
**US**	80%	20%	4310	91%	9%	458
**All**	79%	21%	4987	94%	6%	1038

Table S2. Characteristics of the clinical outcomes analysis cohorts, by PD modality and country among patients on PD ≤ 3 months at study enrollment

	A/NZ		Canada		Japan		South Korea		UK		US	
	CAPD	APD	CAPD	APD	CAPD	APD	CAPD	APD	CAPD	APD	CAPD	APD
Number of patients	66	84	92	228	201	155	121	72	68	106	739	7580
**Demographics**												
Time on PD, months	3.5 [3.3,3.8]	3.5 [3.2,3.7]	3.5 [3.3,3.8]	3.5 [3.3,3.8]	3.5 [3.3,3.7]	3.5 [3.3,3.8]	3.6 [3.4,3.8]	3.6 [3.3,3.8]	3.6 [3.3,3.8]	3.5 [3.3,3.8]	3.5 [3.3,3.8]	3.6 [3.3,3.9]
Years of ESKD	0.32 [0.29,0.34]	0.31 [0.28,0.34]	0.31 [0.28,0.34]	0.31 [0.28,0.33]	0.35 [0.33,0.38]	0.35 [0.32,0.37]	0.54 [0.38,3.35]	0.67 [0.49,10.9]	0.31 [0.28,0.33]	0.30 [0.28,0.33]	0.39 [0.30,1.38]	0.57 [0.33,1.80]
Age, years	58.8 (16.2)	59.5 (14.1)	61.3 (14.9)	60.3 (15.0)	66.7 (13.8)	59.3 (13.5)	54.8 (13.3)	54.0 (12.1)	61.3 (13.9)	57.5 (15.2)	60.4 (14.8)	60.4 (15.1)
Male, %	67%	73%	55%	69%	67%	73%	64%	75%	61%	67%	50%	57%
Black race, %	0%	0%	2%	4%	0%	0%	0%	0%	9%	5%	18%	23%
APD wet day, %	0%	50%	-	58%	67%	36%	0%	7%	-	38%	24%	40%
Employed, %	21%	34%	24%	30%	38%	57%	42%	50%	30%	42%	30%	30%
Systolic blood pressure, mmHg	144 (25.0)	137 (22.1)	133 (17.9)	135 (21.5)	136 (22.3)	144 (21.1)	129 (20.7)	134 (20.9)	139 (24.0)	144 (21.8)	142 (23.1)	140 (24.1)
Caregiver(s) involved in PD exchanges, %	9%	18%	17%	20%	18%	12%	6%	11%	12%	24%	13%	15%
**Comorbidities**												
Coronary artery disease	27%	23%	31%	25%	15%	13%	14%	17%	17%	24%	14%	14%
Congestive heart failure	14%	10%	13%	15%	16%	21%	10%	13%	3%	7%	9%	7%
Cerebrovascular disease	6%	7%	11%	14%	11%	13%	5%	7%	6%	8%	3%	2%
Peripheral vascular disease	15%	15%	18%	15%	9%	5%	5%	7%	8%	13%	3%	3%
Other cardiovascular disease	14%	16%	16%	19%	15%	12%	5%	10%	8%	14%	12%	7%
Diabetes	53%	48%	46%	57%	46%	45%	53%	58%	33%	34%	48%	61%
Hypertension	92%	89%	96%	92%	92%	95%	92%	94%	67%	76%	67%	65%
Cancer (non-skin)	8%	10%	14%	12%	11%	5%	6%	7%	9%	9%	4%	2%
Gastrointestinal bleeding	3%	4%	3%	4%	1%	3%	0%	0%	0%	1%	2%	1%
Lung disease	9%	10%	8%	8%	2%	1%	0.80%	1%	3%	3%	3%	2%
Neurologic disease	3%	4%	2%	3%	6%	6%	0%	1%	2%	1%	1%	1%
Any psychiatric disorder	6%	9%	13%	12%	2%	3%	4%	1%	6%	4%	24%	21%
Recurrent cellulitis/gangrene	3%	1%	2%	2%	0.50%	1%	0.90%	0%	0%	0%	0.80%	0.60%

Transplant waitlist, %	51%	52%	48%	49%	13%	28%	72%	78%	65%	65%	48%	49%
**Lab Values**												
Serum phosphorus, mg/dL	5.5 (1.3)	5.4 (1.3)	4.8 (1.1)	5.2 (1.3)	5.0 (1.3)	5.2 (1.0)	4.7 (1.1)	5.2 (1.4)	4.8 (1.9)	5.0 (1.4)	5.2 (1.6)	5.6 (1.7)
Hemoglobin, g/dL	11.1 (1.8)	11.1 (1.8)	11.0 (1.4)	10.9 (1.4)	11.2 (1.4)	10.9 (1.5)	10.6 (1.6)	10.2 (1.2)	11.0 (1.1)	11.6 (1.5)	11.1 (1.5)	11.0 (1.5)
Serum albumin, g/dL	3.2 (0.6)	3.2 (0.5)	3.4 (0.5)	3.4 (0.5)	3.3 (0.5)	3.3 (0.5)	3.7 (0.5)	3.7 (0.4)	3.3 (0.5)	3.4 (0.5)	3.6 (0.5)	3.6 (0.5)
Serum potassium, mEq/L	4.4 (0.6)	4.5 (0.5)	4.1 (0.6)	4.3 (0.6)	4.2 (0.8)	4.3 (0.7)	4.3 (0.8)	4.4 (0.7)	4.5 (0.7)	4.4 (0.6)	4.3 (0.7)	4.2 (0.6)
Serum creatinine mg/dL	7.3 (2.4)	7.3 (2.6)	6.1 (2.0)	7.1 (2.8)	7.5 (2.4)	8.6 (2.9)	7.6 (3.0)	8.6 (3.6)	6.6 (2.0)	7.2 (2.6)	7.3 (3.6)	8.1 (3.9)
24-hour urine volume, L	1.1 [0.7,1.7]	1.1 [0.8,1.7]	1.1 [0.9,1.5]	1.1 [0.7,1.6]	1.0 [0.6,1.5]	1.1 [0.7,1.5]	1.0 [0.6,1.4]	1.2 [0.8,1.7]	1.4 [0.9,1.8]	1.2 [0.7,1.7]	1.1 [0.5,1.6]	0.8 [0.4,1.4]
4-hour Dialysate/Plasma creatinine ratio	0.75 [0.63,0.86]	0.73 [0.61,0.81]	0.77 [0.70,0.81]	0.73 [0.65,0.81]	0.68 [0.58,0.77]	0.64 [0.55,0.71]	0.69 [0.60,0.74]	0.73 [0.65,0.78]	0.79 [0.72,0.86]	0.72 [0.58,0.80]	0.68 [0.58,0.76]	0.63 [0.54,0.71]
**Dialysis Prescription Details**												
Icodextrin use, %	37%	36%	66%	54%	45%	27%	24%	1%	57%	32%	8%	3%
Icodextrin and high glucose solution, %	19%	18%	25%	17%	10%	4%	8%	0%	6%	5%	7%	12%
Prescribed therapy volume, L	42%	38%	71%	57%	63%	48%	24%	3%	65%	36%	26%	4%
Nutrineal, %	63%	19%	13%	4%	97%	99.30%	45%	75%	14%	30%	1%	0.30%
High calcium, %												
Neutral pH low GDP, %	80%	72%	56%	60%	41%	13%	25%	40%	45%	41%	45%	59%
Use of hypertonic glucose	3%	5%	14%	7%	0%	0.70%	19%	6%	2%	1%	48%	39%
Use of 2.27% but not 3.86%	17%	23%	30%	33%	59%	86%	55%	54%	54%	58%	7%	3%
Use of any 3.86%	37%	36%	66%	54%	45%	27%	24%	1%	57%	32%	8%	3%
Without any 2.27% or 3.86% use	19%	18%	25%	17%	10%	4%	8%	0%	6%	5%	7%	12%
**CAPD connection system**												
Baxter Twin Bag System	37%	83%	88%	88%	0%	0%	0.80%	0%	56%	100%	44%	36%
Baxter Disconnect Y System	0%	0%	1%	4%	0%	0%	0%	0%	28%	0%	24%	18%
Fresenius Stay Safe System (Twin Bag System)	62%	17%	9%	8%	0%	0%	47%	25%	3%	0%	24%	36%
Terumo CAPDEAL TSCD (Heat Welding Automated Connection System)	0%	0%	0%	0%	20%	33%	0%	0%	0%	0%	0%	0%
JMS ZERO System (Twin Bag System)	0%	0%	0%	0%	6%	0%	4%	25%	0%	0%	0%	0%
JMS TEDETAN (Assisted Connection System)	0%	0%	0%	0%	1%	0%	0%	0%	0%	0%	0%	0%
Fresenius Stay Safe System (with drainage bag)	0%	0%	0%	0%	2%	0%	0%	0%	0%	0%	0%	0%
Baxter Twin Bag System (Luer Lock)	0%	0%	0%	0%	8%	0%	45%	50%	0%	0%	0%	0%

Baxter UV Twin Bag System (UV System)	0%	0%	0%	0%	47%	50%	0%	0%	0%	0%	0%	0%
Baxter Disconnect Y system (UV System)	0%	0%	0%	0%	0.50%	0%	0%	0%	0%	0%	0%	0%
Baxter Disconnect Y system (Luer Lock) Bag connection spike system	0%	0%	0%	0%	1%	17%	0%	0%	0%	0%	0%	0%
Terumo Screw Lock System	0%	0%	0%	0%	13%	0%	0%	0%	0%	0%	0%	0%
Others	2%	0%	1%	0%	2%	0%	3%	0%	13%	0%	7%	9%
**Cycler connection system**												
Baxter Twin Bag System		24%		47%		0%		0%		7%		5%
Baxter Disconnect Y System		8%		19%		0%		0%		55%		9%
Baxter Clean Flash Auto (UV Flash Automated Connection System)		0%		2%		0%		0%		0%		10%
Fresenius Stay Safe System (Twin Bag System)		10%		0.70%		0%		4%		7%		4%
Fresenius Sleep Safe Set Plus		15%		0%		0%		17%		0%		0%
Baxter Luer Lock		0%		0%		3%		0%		0%		0%
Baxter UV system		0%		0%		60%		0%		0%		0%
Baxter Spike system		0%		0%		3%		0%		0%		0%
Terumo Screw Lock System		0%		0%		7%		0%		0%		0%
Terumo TSCD system		0%		0%		25%		0%		0%		0%
JMS APD PD-mini Zero		0%		0%		1%		0%		0%		0%
Baxter HomeChoice Autmated PD set with Cassette		5%		0%		0%		75%		0%		0%
Others		39%		31%		0.70%		4%		31%		73%

Results shown as mean (SD), median [IQR] and proportion

(Underlying data for figure above)

Outcome	Subgroup	n Pts	n Events	HR (95% CI)	Interaction *p*-value
Mortality	A/NZ, Canada, UK	642	62	0.75(0.43-1.30)	0.02
	Japan	355	37	0.25(0.09-0.65)	
	South Korea	190	25	0.71(0.25-2.06)	
	US	8276	862	0.96(0.72-1.29)	
	All	9463	986	0.82(0.66-1.03)	
HDT	A/NZ	642	127	0.93(0.61-1.42)	0.94
	Japan	355	90	0.86(0.50-1.46)	
	South Korea	190	30	1.13(0.59-2.17)	
	US	8276	1646	0.94(0.79-1.12)	
	All	9463	1893	0.96(0.83-1.11)	
Mortality/HDT	A/NZ	642	189	0.90(0.63-1.28)	0.28
	Japan	355	127	0.73(0.46-1.15)	
	South Korea	190	55	0.87(0.54-1.40)	
	US	8276	2507	0.95(0.82-1.10)	
	All	9463	2878	0.91(0.81-1.03)	
Peritonitis	A/NZ	612	138	0.72(0.50-1.03)	0.87
	Japan	345	84	0.69(0.39-1.22)	
	South Korea	186	19	1.17(0.32-4.22)	
	US	7980	1236	0.86(0.71-1.04)	
	All	9123	1477	0.83(0.72-0.97)	

Table S3. Adjusted hazard ratios of APD vs. CAPD for clinical outcomes, by subgroups; underlying data for **Figure 4**

Outcome	Subgroup	n Pts	n Events	HR (95% CI)	Interaction *p*-value
Mortality	Assist PD(-)	1187	90	0.84 (0.58-1.23)	0.004
	Assist PD(+)	218	61	0.24 (0.11-0.55)	
	Icodextrin(-)	8830	909	0.89 (0.69-1.15)	0.04
	Icodextrin(+)	633	77	0.53 (0.27-1.05)	
	RKF(-)	1444	241	0.73 (0.45-1.19)	0.43
	RKF(+)	8019	745	0.87 (0.68-1.13)	
	APD dry day	4754	487	0.80(0.62-1.02)	0.56
	APD wet day	6045	677	0.86(0.66-1.11)	
HDT	Assist PD(-)	1187	259	1.01 (0.77-1.33)	0.22
	Assist PD(+)	218	32	0.96 (0.23-3.94)	
	Icodextrin(-)	8830	1752	0.95 (0.81-1.12)	0.82
	Icodextrin(+)	633	141	0.92 (0.59-1.44)	
	RKF(-)	1444	341	1.36 (0.82-2.28)	0.14
	RKF(+)	8019	1552	0.92 (0.79-1.07)	
	APD dry day	4754	828	0.96 (0.77-1.19)	0.04
	APD wet day	6045	1324	0.40 (0.22-0.73)	
Mortality/HDT	Assist PD(-)	9463	2878	0.86(0.75-0.99)	0.14
	Assist PD(+)	9463	2878	0.97(0.84-1.13)	
	Icodextrin(-)	8830	2660	0.93 (0.81-1.07)	0.25
	Icodextrin(+)	633	219	0.81 (0.56-1.18)	
	RKF(-)	1444	582	1.03 (0.72-1.46)	0.69
	RKF(+)	8019	2296	0.90 (0.79-1.02)	
	APD dry day	4754	1315	0.90(0.76-1.07)	0.15
	APD wet day	6045	2000	1.03(0.87-1.22)	
Peritonitis	Assist PD(-)	1143	247	0.86 (0.66-1.12)	0.12
	Assist PD(+)	210	38	0.40 (0.14-1.20)	
	Icodextrin(-)	8527	1339	0.84 (0.71-1.00)	0.87
	Icodextrin(+)	596	138	0.78 (0.52-1.17)	
	RKF(-)	1378	265	1.38 (0.81-2.36)	0.12
	RKF(+)	7745	1212	0.78 (0.66-0.92)	
	APD dry day	4720	700	0.80(0.67-0.96)	0.40
	APD wet day	6000	1022	0.87(0.73-1.04)	

Models were adjusted for time on dialysis, age, sex, Black race, 13 comorbidities (coronary artery disease, cancer [non-skin], other cardiovascular disease, cerebrovascular disease, congestive heart failure diabetes, gastrointestinal bleeding, hypertension, lung disease, neurologic disease, psychiatric disorder, peripheral vascular disease, and recurrent cellulitis/gangrene), albumin, creatinine, 24 hour urine volume, potassium, icodextrin, transplant waitlist status, assisted PD, facility size; stratified by country; models accounted for facility clustering using a robust sandwich estimator.

Table S4. Adjusted hazard ratios of APD vs. CAPD for clinical outcomes, by 4-hour dialysate/plasma creatinine ratio

Outcome	Dialysate/Plasma Creatinine Ratio[a]	n Pts	n Events	HR (95% CI)	Interaction *p*-value
Mortality	≤0.55[b]	663	44	-	
	0.56-0.64	505	46	0.99(0.36,2.75)	0.4
	0.65-0.80	756	79	0.68(0.35,1.32)	
	≥0.81	259	28	0.40(0.16,1.04)	
HDT	≤0.55	508	90	0.88(0.40,1.89)	0.7
	0.56-0.64	505	97	1.47(0.66,3.25)	
	0.65-0.80	756	146	0.88(0.54,1.44)	
	≥0.81	259	65	0.95(0.49,1.84)	
Mortality/HDT	≤0.55	508	122	1.12(0.51,2.43)	0.5
	0.56-0.64	505	143	1.31(0.68,2.51)	
	0.65-0.80	756	224	0.83(0.55,1.26)	
	≥0.81	259	93	0.74(0.42,1.30)	
Peritonitis	≤0.55	493	60	0.51(0.25,1.06)	0.3
	0.56-0.64	485	84	1.04(0.49,2.20)	
	0.65-0.80	722	136	1.05(0.67,1.66)	
	≥0.81	252	50	0.70(0.35,1.43)	

a. Dialysate creatine ratio at 4 hours on the PET test was available for 2028 of patients (21% of the overall study population).
Models were adjusted for time on dialysis, age, sex, Black race, 13 comorbidities (coronary artery disease, cancer [non-skin], other cardiovascular disease, cerebrovascular disease, congestive heart failure diabetes, gastrointestinal bleeding, hypertension, lung disease, neurologic disease, psychiatric disorder, peripheral vascular disease, and recurrent cellulitis/gangrene), albumin, creatinine, 24 hour urine volume, potassium, transplant waitlist status, facility size; stratified by country; models accounted for facility clustering using a robust sandwich estimator.

b. There were no CAPD patients with Dialysate creatine ratio ≤ 0.55 with a death event

Table S5. Adjusted hazard ratios of APD vs. CAPD for clinical outcomes censoring follow-up at PD modality switch

	Main results (intent to treat)			Censoring at PD modality switch		
**Outcome**	**n Pts**	**n Events**	**AHR(95%CI)**	**n Pts**	**n Events**	**AHR(95%CI)**
Mortality	9463	986	0.82(0.66-1.03)	9463	930	0.82(0.63-1.06)
HDT	9463	1893	0.96(0.83-1.11)	9463	1777	1.00(0.86-1.18)
Mortality/HDT	9463	2878	0.91(0.81-1.03)	9463	2706	0.94(0.83-1.07)
Peritonitis	9123	1477	0.83(0.72-0.97)	9123	1388	0.88(0.74-1.05)

Separate models were conducted for each outcome/column. N=568 (6%) of patients experienced a modality switch during follow-up; 63% were from CAPD to APD.

Models were adjusted for time on dialysis, age, sex, Black race, 13 comorbidities (coronary artery disease, cancer [non-skin], other cardiovascular disease, cerebrovascular disease, congestive heart failure diabetes, gastrointestinal bleeding, hypertension, lung disease, neurologic disease, psychiatric disorder, peripheral vascular disease, and recurrent cellulitis/gangrene), albumin, creatinine, 24 hour urine volume, potassium, icodextrin, transplant waitlist status, assisted PD, facility size; stratified by country; models accounted for facility clustering using a robust sandwich estimator.
